# Ramf: An Open-Source R Package for Statistical Analysis and Display of Quantitative Root Colonization by Arbuscular Mycorrhiza Fungi

**DOI:** 10.3389/fpls.2019.01184

**Published:** 2019-09-27

**Authors:** Marco Chiapello, Debatosh Das, Caroline Gutjahr

**Affiliations:** ^1^Institute for Sustainable Plant Protection, CNR, Torino, Italy; ^2^Faculty of Biology, Genetics, LMU Munich, Martinsried, Germany; ^3^Plant Genetics, TUM School of Life Sciences Weihenstephan, Technical University of Munich (TUM), Freising, Germany

**Keywords:** R, data display, arbuscular mycorrhiza (AM), root colonization, statistics

## Abstract

Data analysis and graphical representation form an essential part of scientific research dissemination. The life-science community is moving towards a more transparent presentation of single data points or data distributions and away from mean values displayed as bar charts. To facilitate transparent data display to the mycorrhiza community, we present “Ramf” an open-source R package for statistical analysis and preparation of a variety of publication-ready plots, custom-made for analyzing and displaying quantitative root colonization by arbuscular mycorrhiza fungi or any kind of data to be displayed in the same format. Ramf replaces the scripting needed for data analysis and can be readily used by researchers not acquainted with R. In addition, the package is open to improvements by the community. Ramf is available at https://github.com/mchiapello/Ramf.

## Introduction

Arbuscular mycorrhiza (AM) is an ancient mutualistic association between arbuscular mycorrhiza fungi (AMF) of the phylum *Glomeromycotina* and approximately 80% of land plant species (Parniske, 2008; Spatafora et al., 2016). The development and function of this symbiosis is investigated by an active research community because of its fascinating biology and because the fungus confers increased mineral nutrition and stress resistance to plants (Smith and Smith, 2011; Gutjahr and Parniske, 2013; Chen et al., 2018). During root colonization, the fungus first attaches to the root surface *via* a hyphopodium, it then enters the root forming intraradical hyphae and subsequently highly branched arbuscules, which are crucial for nutrient exchange between the symbionts, and vesicles, thought to serve as fungal carbon store, in the root cortex (Gutjahr and Parniske, 2013; Choi et al., 2018). An estimation of quantitative total colonization of the root system, and the frequency of hyphopodia, intraradical hyphae, arbuscules, and vesicles is integral to phenotyping arbuscular mycorrhiza development in wild type and mutant plants, and to correlating fungal root colonization with symbiotic function (Montero et al., 2019). In addition, and especially for plant mutants with defects in inducing or supporting hyphopodia formation, also the extraradical hyphae, which have germinated from spores and linger on the root surface, are quantified (Gutjahr et al., 2015; Roth et al., 2018).

Two methods are primarily used by AM researchers to score these fungal structures and to thereby estimate the root colonization level. For the gridline intersect method, root intersections are scored for presence or absence of fungal structures, and % root length colonization is calculated based on the proportion of intersection counts containing fungal structures to total intersection counts (Giovannetti and Mosse, 1980; McGonigle et al., 1990). The method, based on Trouvelot et al. (1986), scores the frequency and intensity of colonization based on the observation of a number of root pieces with defined size (e.g., 1 cm). The frequency of colonization is calculated as the number of colonized root pieces to the total number of visualized root pieces. The intensity of colonization is recorded by classifying observed root pieces from 1 to 5 by density and coverage of colonization, as well as density and coverage with individual fungal structures, with 1 representing very low colonization, and 5 representing full colonization of the root piece. The scores are then used to calculate a percentage for intensity of colonization as well as individual fungal structures.

Data describing quantitative AM colonization of roots need to be statistically analyzed and visualized with clear graphical display. With the advent of numerous data analysis platforms, statistical analysis and graphical representation have been made easy. However, most of the easy-to-use packages, such as Graphpad Prism, SigmaPlot, SPSS, SAS, BioVinci, XLStat, Matlab, and many others, have complex user interfaces, are costly, platform-specific, and require frequent renewal of the subscription. On the other hand, open-source softwares, such as R and Python, require some scripting knowledge to operate the various packages for analysis and plotting of the data. Even with pre-assembled packages, most data sets require tweaking of the scripts based on the data structure to obtain uniformly drawn plots across different data inputs. To facilitate analysis and display of quantitative AM fungal root colonization data, we present an R package, named Ramf (R for Arbuscular Mycorrhiza Fungi), which requires only specifically formatted input data (based on the counting method). The user simply needs to install the package in R and follow the scripts according to the instructions in the Ramf manual. Excellent usability (minimal scripting, easy, and consistent data output) and robust high-quality output (multiple plot types) are two great advantages of Ramf for the elaboration of AM colonization data to obtain statistical summary and publication-ready plots in a short time.

## Materials and Methods

### Plant Growth, Fungal Inoculation, and Quantification of Root Length Colonization

For the phosphate dose-response experiment *Lotus japonicus* ecotype Gifu seedlings were germinated on 1% water-agar for 3 days in the dark. Then they were moved to a long day photoperiod (16h L/8h D). At the age of 2 weeks the *Lotus japonicus* seedlings were inoculated with 500 spores per plant of *Rhizophagus irregularis* DAOM197198 and subsequently grown in three different growth substrates: sand, sand + Terragreen (Attapulgite clay; OilDry, UK) and sand + calcined clay at three different phosphate concentrations: 2.5 µM (Low phosphate), 250 µM (Medium phosphate), and 2500 µM (High phosphate). Fifteen milliliters of half-Hoagland media containing the indicated concentration of phosphate was provided three times a week to each pot. Plant roots were harvested at 6 weeks post-inoculation (wpi), stained with acid ink (Vierheilig et al., 1998), and scored for fungal structures using the gridline intersection method (McGonigle et al., 1990).

The data of the strigolactone experiment have been previously published in Kountche et al. (2018) and are used here for illustrating the functionalities of Ramf regarding the Trouvelot quantification method (Trouvelot et al., 1986). The method for data acquisition is described in Kountche et al. (2018).

### Ramf Manual

#### Data Preparation, Inspection, and Management in Ramf

Data collected from microscopic visualization is ready for analysis, independent of the scoring system. Data should be prepared in a specific format prior to input into Ramf package. For both scoring systems, data sets contain samples and replicates in the first two columns. The first column contains the sample names, and all replicates of a treatment or genotype should be provided with the correct same sample name since Ramf will treat all samples with misspelled names as different treatments/genotypes. These sample names will also feature in the tables and plots and should therefore be immediately designed to be presentable and easy to understand by the reader. The first treatment/genotype (represented by a set of replicates) is always treated as control. This will be important when Ramf adds the statistical analysis to the plot. The replicate column contains the indication of the replicates (alphabetical letters or numerical values are preferred). If two samples have the same sample name and same replicate name, Ramf will treat the replicates as “technical” replicates, whereas if the sample name is the same, but the replicate name is different, they will be treated as biological replicates. Ramf can handle different numbers of technical and biological replicates.

For the gridline intersect method there are five more columns: total, hyphopodia, intraradical hyphae (IntrHyphae), arbuscules, vesicles. At this stage, the user cannot specify other or additional column names, but Ramf can handle missing columns. Furthermore, Ramf is open to improvements and additional columns can be added in the future. The user can report only the columns needed but should always report information for the two first columns, whereas none of the other ones is strictly required (for example, samples, replicates, arbuscules, vesicles).

For the Trouvelot method, besides the first two columns reporting sample name and replicate, there is a third column called ‘scoring’, which contains the colonization score (e.g., 1A3, 5A3, 1A2, …).

**Figure 1:**
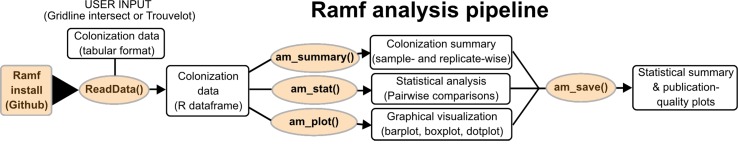
Ramf workflow. Ramf functions are shown in orange boxes and the purpose of the functions in white boxes.

After table preparation, the data can be read into R using the function: “readData()” (**Figure 1**). The first operation done by the function is the quality check of the input data set: (i) the data set should have correct dimensions, (ii) column names should be labeled as mentioned above, (iii) data should not contain NAs, (iv) for the Trouvelot method Ramf checks whether all scores are valid. If all these conditions are met, the data are read into R, otherwise a warning is thrown in R console, which can then be addressed by inputing the data correctly.

As shown in **Figure 1**, once read as a dataframe in R, a data summary can be obtained using the function “am_summary()”. This function summarizes the data in a tabular format and also computes the scoring values (F, M, a, A) for the Trouvelot scoring system. The function reports two tables: the first one combines the data for technical replicates of each biological replicate of each sample and summarizes the data for biological replicates of each sample, whereas the second one presents the sample-wise inferential statistics for the data. The columns of the summary tables report the mean and the standard error for each variable (AM fungal structure in the gridline or score in the Trouvelot scoring system).

#### Statistical Analysis Methods

Subsequently, the function “am_stat()” performs the statistical analysis on the input data set. The function uses the Kruskal–Wallis test (Kruskal and Wallis, 1952), a non-parametric statistical method based on median comparison between two sample groups. This method is meant to test whether samples belong to the same distribution. Ramf package uses this statistical test because it does not assume a normal distribution, as normal distribution is usually not met in data reporting quantitative root colonization by AM fungi. The *post hoc* test is using the criterion of Fisher’s least significant difference.

All *p* values resulting from the comparisons are variable dependent, which means that Ramf tests total colonization, arbuscules, and other fungal structures separately.

Additional functions for statistical comparison are: “am_anova_grid()” or “am_anova_trouvelot(),” for one-way ANOVA; and am_2anova_grid() and am_2anova_trouvelot() for two-way ANOVA. The functions for one way ANOVA are non-parametric and use the Kruskal–Wallis test, as per am_stat(). The 2-way ANOVA is parametric and provides two plots to check for ANOVA assumptions. The first is a residual versus fitted plot to check whether there is equal variance (homoscedasticity). The second is a Q-Q plot to check whether the data are normally distributed.

#### Plot Methods and Plot Statistics

To visualize the data, Ramf package provides three plot types: (i) Dotplot, to be used with few data points (“am_dotplot()”), (ii) Barplot, to be used with a larger number of data points (“am_barplot()”), and (iii) Boxplot to be used with a large number of data points (“am_boxplot()”). In Ramf, with one simple R function, you can create ready-to-publish plots. Plots are completely customizable and users can define colors, titles, legends, plot theme, and statistics. The latter is particularly important because statistics can be displayed on the plot in two different ways: 1) asterisks, which compare the control to the treated samples or wild type to mutant genotypes etc with a default or user-defined *p* value cut-off or 2) letters, which group the treatments/genotypes that belong to the same distribution.

#### Export Data

The last fundamental step is the data export in a ready-to-publish format. Ramf provides a unique function to save all statistical and graphical outputs: “am_save()”. The user can save the summary tables, the statistical analysis and the plot, defining dimensions, resolution, and format. The choice for saving the output as a table or plot is made automatically by the function. The default format for saving the tabular data is comma separated value (csv), in order to preserve the highest compatibility in all the operating system (OS), the plot can be saved in several formats and resolutions.

## Results and Discussion

### Ramf Workflow

The first operation is to install R and RStudio on your computer. R can be installed from the CRAN website (The Comprehensive R Archive Network, https://cran.r-project.org/) and RStudio, an IDE (integrated development environment) for R, which can be downloaded for free at the following link: https://www.rstudio.com/. The user should download and install R and RStudio compatible with his/her operating system. After launching RStudio, the first step is to install devtools package followed by Ramf package using the following commands:


> install.packages(“devtools”)
> devtools::install_github(“mchiapello/Ramf”)


The next step is to load Ramf package into the R environment:


> library(“Ramf”)

Ramf workflow requires four steps: (i) data input, (ii) summary or score analysis, (iii) statistical analysis, and (iv) graphical visualization (**Figure 1**). For each step, a specific function has been designed. Each function aims to perform a specific operation, with less options as possible in order to increase user-friendliness. R functions are designed as follows: functionName, open parenthesis, options for the function, and close parenthesis. For example, the first function in the package, called “readData,” takes two options: the first one is the path to the data file and the second one is the scoring method.


> readData(“data/gridData.csv”, type = “grid”)

Here, a general advice is to always use the same directory folder for the input data file and the script utilizing the data or in other words move the input data file to the current working directory, in order to be as tidy as possible and create a shareable project.

In the upcoming sections, a detailed explanation of all the functions and options will be provided utilizing two case studies for analysing root colonization data.

### Case Studies

The aim of this section is to demonstrate the use of Ramf package starting from package installation through data analysis to data export. The complete script will be available as **Supplementary Material**. There are two case studies: one using the gridline scoring system and the second one using the Trouvelot scoring system.

### Case Study 1: Sand Is an Optimal Substrate for High Phosphate-Mediated Inhibition of *Lotus japonicus* Root Colonization by *Rhizophagus irregularis*

AM development can be inhibited by high phosphate fertilization (Breuillin et al., 2010; Balzergue et al., 2010). Plant species differ in their physiological optima and, therefore, in the phosphate concentration required for effective AM inhibition as well as the growth substrate permitting optimal inhibition. In this experiment, we searched for an optimal combination of phosphate concentration and growth substrate, which reliably inhibits root colonization by AM fungi in *Lotus japonicus*. We used three phosphate concentrations and three different substrates: sand alone, sand + terragreen, and sand + calcined clay. Statistical comparison using Ramf am_anova_grid() and am_2anova_grid() functions suggested strongest inhibition of root colonization by 2500 µM phosphate (high phosphate) vs. 2.5 µM phosphate (low phosphate) in sand compared to the other two substrates as suggested by the lowest *p* value obtained for sand in the statistical comparison between the total colonization in the two phosphate conditions (**Figure S1**; total colonization data in **Tables S1**, **S2**, **S3** and **S4**; R code in **Code S1**). **Table S4** used for a 2-way ANOVA analysis was prepared by combining **Tables S1**, **S2** and **S3** and including an extra column “trt” providing information on the substrate being used in the combined data set. Diagnostic plots to check ANOVA assumptions for equal variance (homoscedasticity) and normality (Q-Q plot) are provided in **Figures S2** and **S3**.

Corresponding with root colonization, only plants grown in sand displayed observable growth differences in the three different phosphate levels: Low phosphate (2.5 μM), Medium phosphate (250 μM) and High phosphate (2500 μM) (**Figure S4**), whereas ´there were no differences for the other two growth substrates (data not shown), suggesting that terragreen and calcined clay prevented complete plant-availability of the phosphate. We therefore continue here with the colonization data obtained in sand. Quantification of root length colonization (**Table 1**) and statistical analysis show a dose-dependent inhibition of colonization by phosphate in roots of *Lotus japonicus* grown in sand.

**Table 1:** Input data file for gridline intersect quantification method. The data (in %) are from *Lotus japonicus* roots grown in sand at 2.5 µM (low), 250 µM (medium) and 2500 µM (high) phosphate at 6 wpi with *Rhizophagus irregularis*.

Samples	Replicates	Total	Hyphopodia	IntrHyphae	Arbuscule	Vesicle
Low phosphate	A	88	4	88	78	40
Low phosphate	B	95	4	95	76	35
Low phosphate	C	87	6	87	70	31
Low phosphate	D	74	5	74	62	27
Low phosphate	E	95	3	95	79	25
Low phosphate	F	93	4	93	85	41
Low phosphate	G	80	4	80	59	20
Medium phosphate	A	79	4	79	61	27
Medium phosphate	B	72	3	72	59	20
Medium phosphate	C	52	2	52	40	21
Medium phosphate	D	80	4	80	63	29
Medium phosphate	E	53	2	53	41	15
Medium phosphate	F	63	4	63	49	25
Medium phosphate	G	62	4	62	48	24
High phosphate	A	21	2	21	21	8
High phosphate	B	7	1	7	5	2
High phosphate	C	5	1	5	5	2
High phosphate	D	18	2	18	18	6
High phosphate	E	7	1	7	5	1
High phosphate	F	17	2	17	11	2
High phosphate	G	2	0	2	2	1

The first step is to load the data, formatted as **Table 1** (**Table S5**), into R:


> gr <- readData(“gridData.csv”, type = “grid”)

The readData() function needs two types of information: the path to the input data file and the scoring method. This step assigns the data to a dataframe “gr”. Name variables can be tedious to work with and often they are called ‘x’ or ‘xx’ or ‘xx2’, but we recommend to name your variables in a meaningful way such as “gridline” or “trouvelot”.

The second step is to summarize or compute the colonization scores, by using the summary function:


> grs <- am_summary(gr)

**Figure 2:**
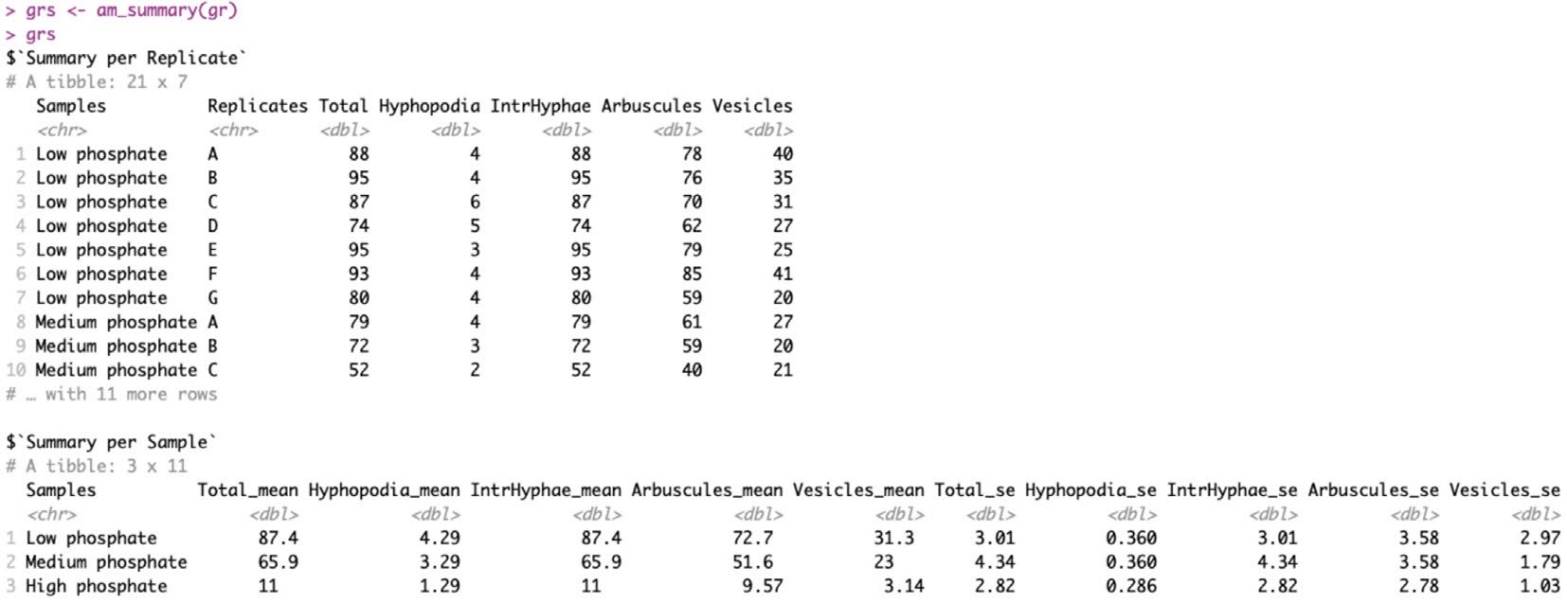
Output of am_summary() function. The output includes two tables: the first one called “Summary per Replicate” summarizes the data if technical replicates are present. The second one called “Summary per Sample” summarizes the data by treatment. The first column reports the sample names, whereas the columns between two and six report the means for each variable. The last five columns show the standard errors.

This function does not take any additional arguments other than the dataframe “gr”, on which to perform the summary. **Figure 2** reports the output of the “am_summary()” function.

In the third step the statistical analysis is performed, using the “am_stat()” function:


> grst <- am_stat(gr)

If the analysis does not need a multiple-correction test, the function works with no argument,


> grst <- am_stat(gr, method = “fdr”)

whereas if the correction test is needed, the user can specify the correction method by choosing between: Holm, Hommel, Hochberg, Bonferroni, Benjamini-Hochberg, Benjamini-Yekutieli, or fdr adjustment of *p* values.

**Figure 3:**
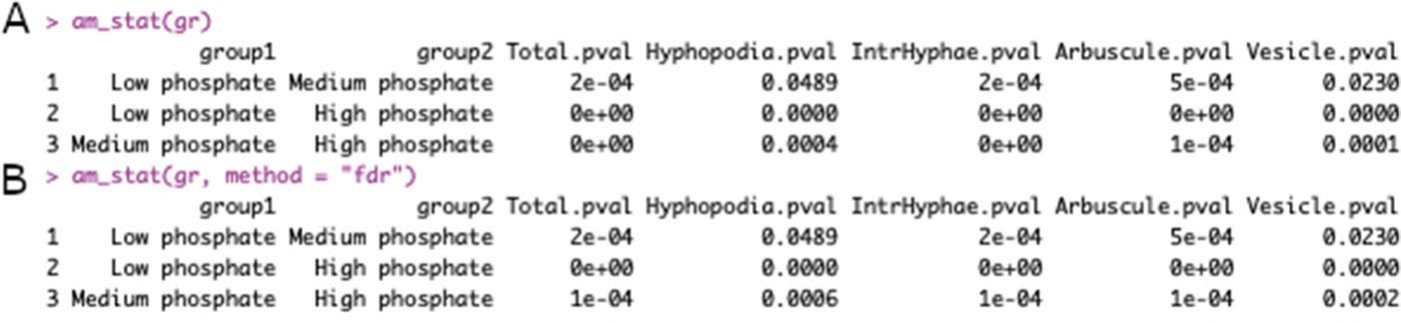
Output of am_stat() function. Default output of am_stat() function **(A)**. Output after Kruskall-Wallis test with “False Discovery Rate” correction **(B)**. The first two columns contain the sample names to be compared, whereas the following columns contain the *p* values for each variable of the data set.

The outputs of the “am_stat()” function, with and without the correction method, are shown in **Figure 3**.

The fourth step is data plotting. We recommend using dotplots or otherwise boxplots for graphically representing AM quantification data. Since the data set contains few replicate samples, dotplot is the best choice in this case. The package provides two different possible data displays.


> am_dotplot(gr)
> am_dotplot2(gr)


**Figure 4:**
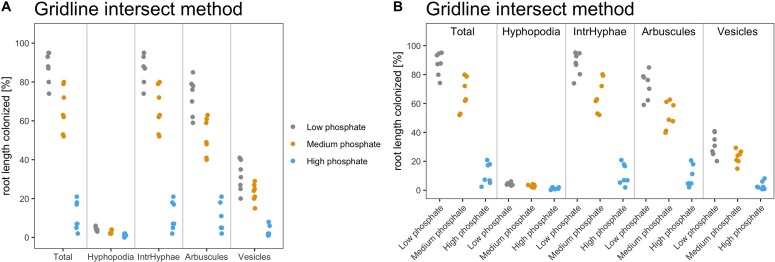
Default output of dotplot() and dotplot2() function. The figure shows the default output of the dotplot() and dotplot2() function. **(A)** The plot is divided into sectors, one per variable, and the variable name is reported at the bottom of each sector. The y-axis shows the percentage of root colonization. Each dot is a biological replicate. **(B)** The variable name is reported on the top of each sector, whereas at the bottom of the x-axis, the treatment (sample name) is reported. Each dot is a biological replicate. Plot titles can be modified according to individual needs.

**Figure 4** shows the default dotplots obtained using our Ramf package; from now on we will use the display shown in **Figure 4A**.

The plot can be customized in different ways as the plotting system is based on the “ggplot2” package. Users need to install and load ggplot2 package before adding extra features to the plots, using the following command:


> install.packages(“ggplot2”)
> library(ggplot2)


**Figure 5:**
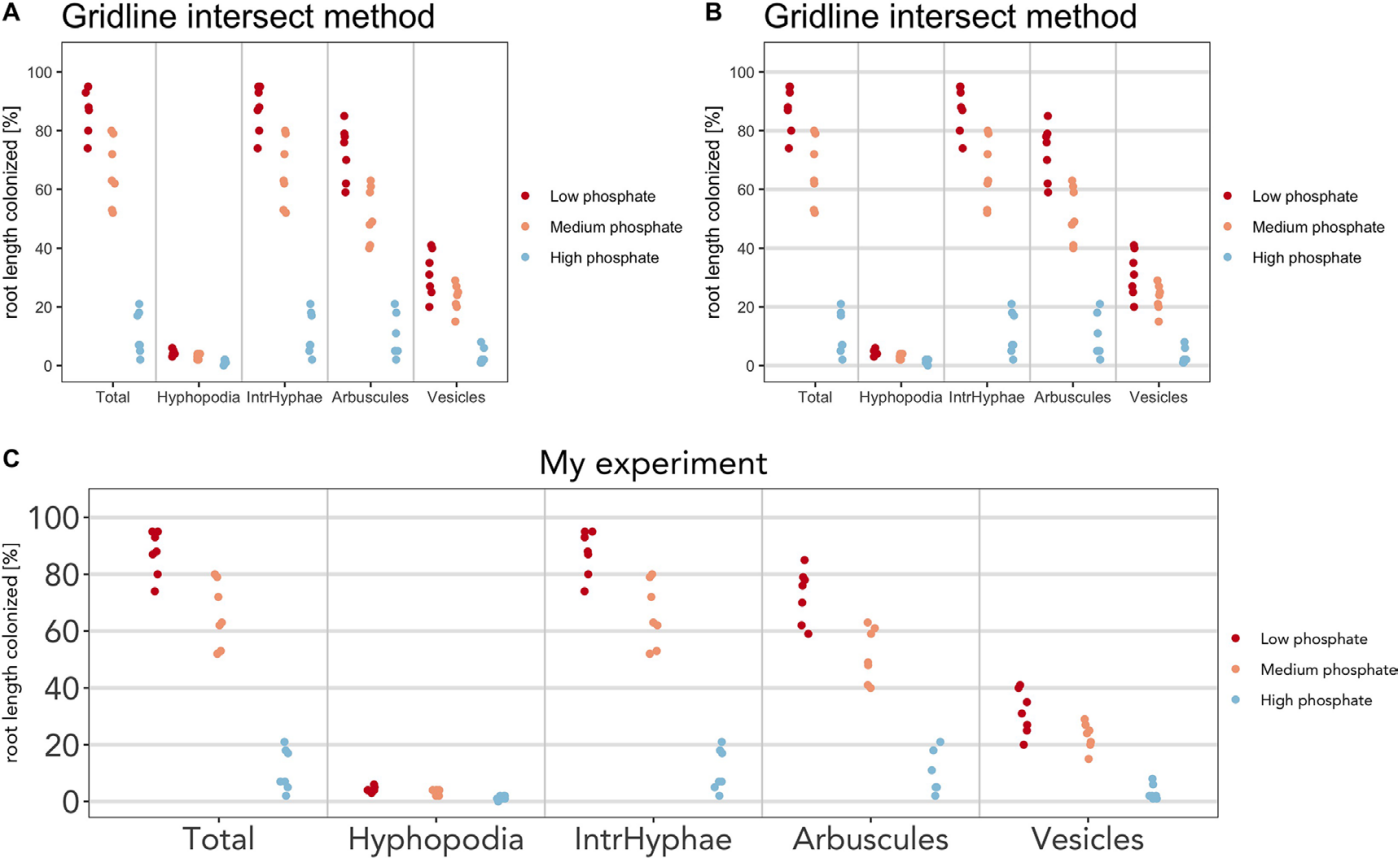
Plot customization. The plot can be customized by the user. **(A)** Plot with different color scheme compared to the default. **(B)** Horizontal lines have been added. **(C)** Font and size of title and x-axis labels have been changed.

For example, below we have shown few modifications to the default plot:

a) The color scheme (**Figure 5A**)


> am_dotplot(gr, cbPalette = c(‘#ca0020’, ‘#f4a582’, ‘#92c5de’))

a) Add horizontal grid to better discriminate the samples (**Figure 5B**)


 > am_dotplot(gr, cbPalette = c(‘#ca0020’, ‘#f4a582’, ‘#92c5de’))+ theme(panel.grid.major.y = element_line(size = 1, colour = “grey90”))

a) Add a title, modify its default position, font of the letters, and the dimension on the text (**Figure 5C**)


> am_dotplot(gr, main = “My experiment”, cbPalette = c(‘#ca0020’, ‘#f4a582’, ‘#92c5de’))+ theme(panel.grid.major.y = element_line(size = 1, colour = “grey90”), plot.title = element_text(hjust = .5), text = element_text(family = “Avenir”), axis.text = element_text(size = 18))


Depending on the operating system (OS), some fonts may not be available, and hence, alternative fonts, such as Sans, can be used in the above code.

Furthermore, the result of statistical analysis can be added to the plot. It is possible to include the statistical results on the plot with the desired correction method (default is “none”) and with a *p* value threshold of 0.05. As mentioned above, users can utilize either asterisks or letters to show the statistical differences between the samples.

1) Asterisks: Asterisks above the dots, bars or boxes show, which sample is significantly different from the control for the respective variable (fungal structure or score type). The control is always the first element of the list.


> am_dotplot(gr, annot = “asterisks”)

To include the statistical correction for the *p* values, the user can select, which correction methods to use.


> am_dotplot(gr, annot = “asterisks”, method = “BH”)

**Figure 6:**
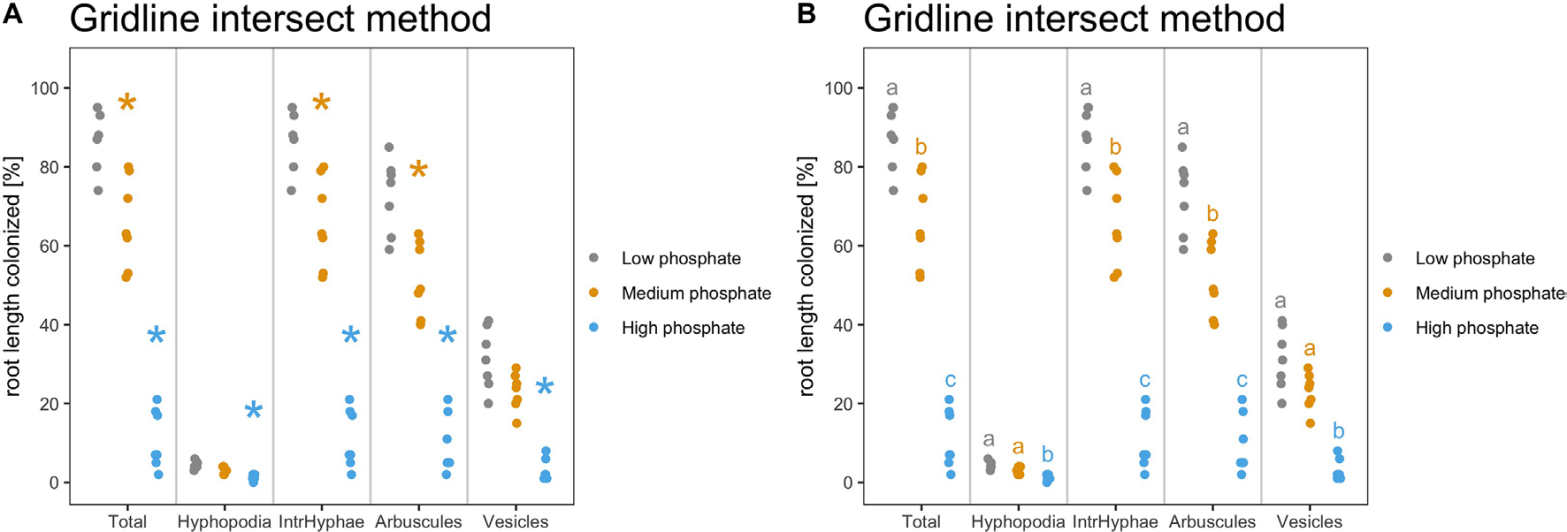
Indication of statistically significant differences on the plot. Strip chart containing indications of statistically significant differences. **(A)** The asterisks compare the treated samples against the control. Plot with Kruskall Wallis test followed by “Bonferroni Hochberg” correction and *p* value threshold set to 0.01. **(B)** Same statistical test as in **(A)** but different letters indicate different statistical groups. **(A, B)** All statistical comparisons have been performed per variable and not across variables to account for the biological meaning of the comparison. For example, the asterisks and letters present in the section “Total” have only a meaning for “Total” and cannot be compared with the asterisks from the other sections.

Finally, it is possible to combine correction method and *p* value threshold (**Figure 6A**).


> am_dotplot(gr, annot = “asterisks”, method = “BH”, alpha = 0.01)

The asterisks allow to graphically display the statistical difference between the control sample and other samples, with a *p* value cutoff of 0.01 rather than the default value of 0.05. Therefore, *p* values lying between 0.05 and 0.01 will not be displayed with asterisks.

2) As an alternative, to highlight the statistical difference between all the samples, Ramf can add “letters” to the plot. Letters group the samples according to their statistical similarity (**Figure 6B**).


> am_dotplot(gr, annot = “letters”, method = “BH”, alpha = 0.01)

**Figure 7:**
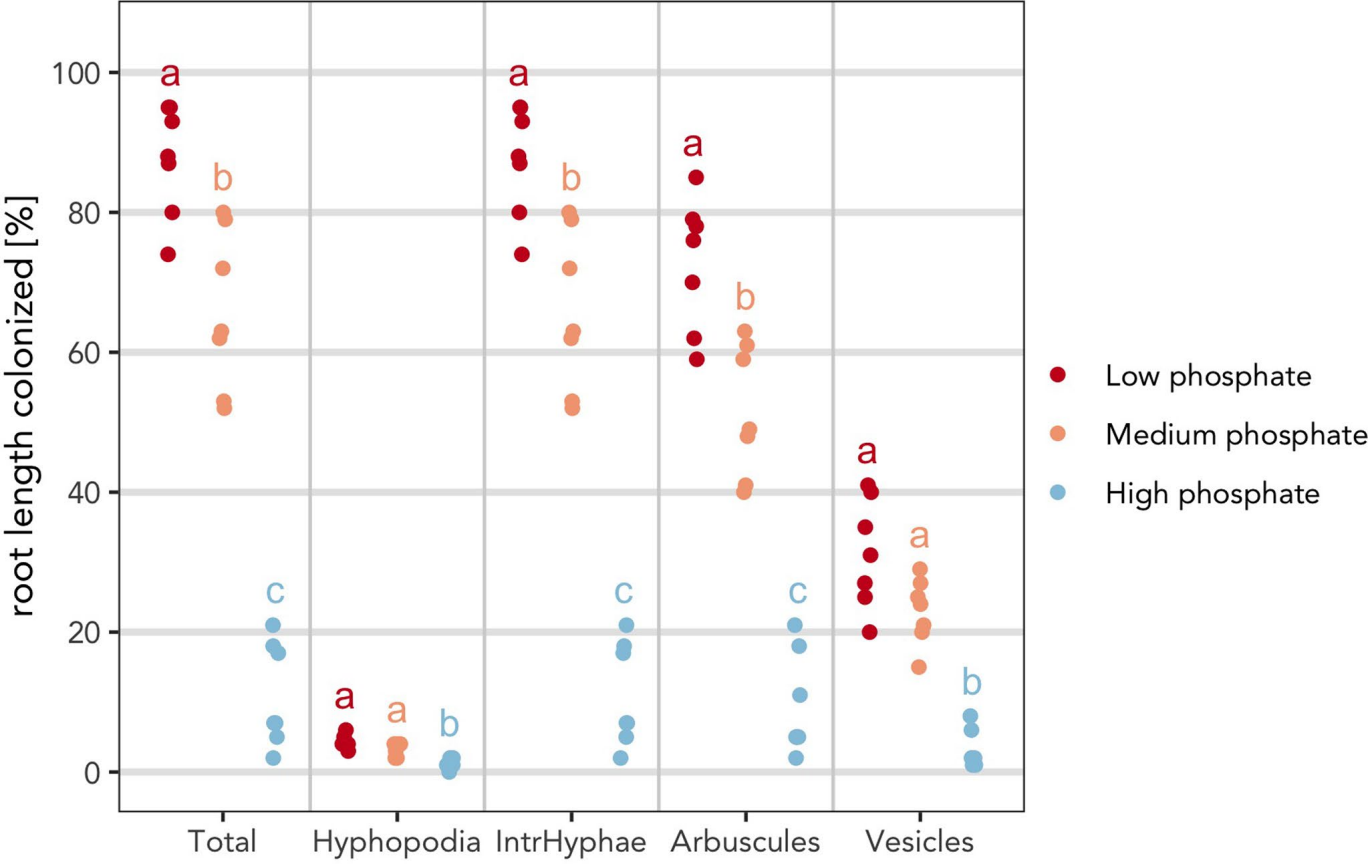
“Complete” plot. The plot combines the statistical analysis and all the features that can be included by the user. Different letters indicate different statistical groups.

Finally, it is possible to combine all the previous adaptations to produce the final plot (**Figure 7**).


> am_dotplot(gr, annot = “letters”, method = “BH”, alpha = 0.01, main = “Grid experiment”, cbPalette = c(‘#ca0020’, ‘#f4a582’, ‘#92c5de’))+ theme(panel.grid.major.y = element_line(size = 1, colour = “grey90”), plot.title = element_text(hjust = .5), text = element_text(family = “Avenir”), axis.text = element_text(size = 16))

The fifth step is to save the statistical tables and the plot. Ramf provides a single function to export everything.

1) To save the summary data:


> am_save(grs, “Exp001”) or > am_save(am_summary(gr), “Exp001”)

The two commands produce the same output: two files named “Exp001_per_Replicate.csv” and “Exp001_per_Sample.csv”.

2) To save the statistical analysis:


> am_save(grst, “Exp001”) or > am_save(am_stat(gr), “Exp001”)

Also, in this case, the two commands produce the same output: a csv file named “Exp001_stat.csv”.

3) To save the plot the “am_save” function can take more options in order to specifically customize the output. It is possible to set the width, the height, the dimension units, and the resolution (dpi) of the plot. Different formats and different resolutions allow the user to produce low-resolution figures for the first manuscript submission and high-resolution figures for the final submission, just by changing a number in the code. Below we have provided a few examples of the export format and resolutions:


> p1 <- am_dotplot(gr, main = “Grid experiment”, cbPalette = c(‘#ca0020’, ‘#f4a582’, ‘#92c5de’), annot = “letters”, method = “BH”)+ theme(panel.grid.major.y = element_line(size = 1, colour = “grey90”), plot.title = element_text(hjust = .5), axis.text = element_text(size = 14))
> am_save(p1, “Exp001.pdf”, width = 21, height = 21, units = “cm”, dpi = 300)
> am_save(p1, “Exp001.jpeg”, width = 10, height = 10, units = “in”, dpi = 72)
> am_save(p1, “Exp001.eps”, width = 210, height = 210, units = “mm”, dpi = 320)
> am_save(p1, “Exp001.svg”, width = 21, height = 21, units = “cm”, dpi = 300)


The complete script is attached as **Code S2**.

### Case Study 2: Effect of the Strigolactone Analogs Methyl Phenlactonoate 1 and 3 on Root Colonization by Arbuscular Mycorrhiza Fungi

Strigolactones (SLs) stimulate the activity of AM fungi and also act as key regulators of plant architecture (Waters et al., 2017). Kountche et al. (2018) investigated the effect of the SL analogs methyl phenlactonoate 1 and 3 (MP1, MP3), in comparison to the widely used SL-analog *rac*-GR24 on rice root colonization by the AM fungus *Funneliformis mossae* and quantified colonization according to Trouvelot et al. (1986). To train Ramf for the Trouvelot scoring method we used part of their data in this case-study. **Table 2** shows a subset of these data. In contrast to the data obtained by gridline intersect scoring (case study 1), this data set also contains technical replicates (**Table S6**).

**Table 2:** Subset of the input data file for arbuscule abundance data for SL-analogs experiment.

Samples	Replicates	Scoring
Control	1	3A3
Control	1	5A3
Control	1	3A3
Control	2	4A3
Control	2	2A3
Control	2	2A3
GR24 10-7M	1	3A3
GR24 10-7M	1	3A3
GR24 10-7M	1	3A3
GR24 10-7M	2	5A3
GR24 10-7M	2	5A3
GR24 10-7M	3	5A3
MP3 10-8M	1	4A3
MP3 10-8M	1	5A3
MP3 10-8M	1	4A3
MP3 10-8M	2	3A2
MP3 10-8M	2	5A3
MP3 10-8M	2	5A3
MP1 10-7M	1	3A2
MP1 10-7M	1	5A2
MP1 10-7M	1	1A2
MP1 10-7M	2	3A2
MP1 10-7M	2	2A3
MP1 10-7M	2	2A3

**Figure 8:**
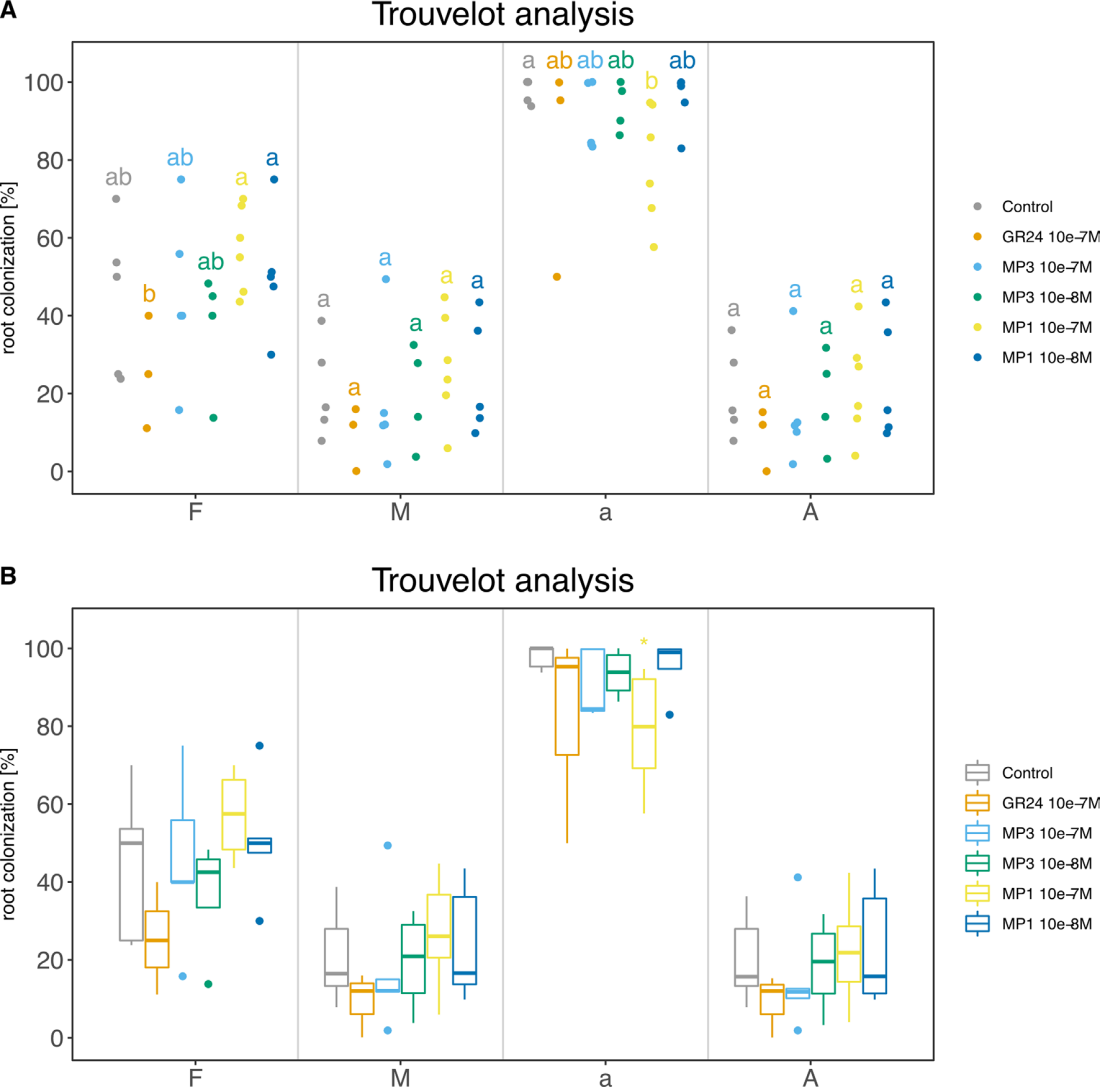
Trouvelot analysis method. The two plots display the same data in two different flavors: **(A)** strip chart with letters to indicate statistical groups and **(B)** box-whisker plot with asterisks to indicate statistical difference compared to the control. Bold black line, median; box, interquartile range; whiskers highest and lowest data point within 1.5 interquartile range; dots, outliers outside the 1.5 times the interquartile range. **(A, B)** Compounds used: GR24; MP (Methyl phenlactonoate) 1 and 3. Kruskall-Wallis test with no correction method and p < 0.05 was used to assess statistical differences between samples.

For this case study, we report all commands used to produce the final summary data (**Code S3**). Final plots are shown as dotplot and boxplot (**Figure 8**).


# Load libraries
library(Ramf)
library(ggplot2)
# Read data in
tr <- readData(“trouvelotData.csv”, type = “trouvelot”)
# Summar
trs <- am_summary(tr)
# Statistics
trst <- am_stat(tr, method = “fdr”)
# Plots
p1 <- am_dotplot(tr, main = “Trouvelot experiment”, annot = “letters”)+ theme(plot.title = element_text(hjust = .5), axis.text = element_text(size = 14))
p2 <- am_boxplot(tr, main = “Trouvelot experiment”, annot = “letters”)+ theme(plot.title = element_text(hjust = .5), axis.text = element_text(size = 14)) + geom_jitter(width = 0.1, colour = “black”, alpha = 0.3)
# Save
## Summary
am_save(trs, “Exp001”)
## Statistics
am_save(trst, “Exp001”)
## Plot
am_save(p1, “Exp001.jpeg”, width = 29, height = 21, units = “cm”, dpi = 300)
am_save(p2, “Exp002.jpeg”, width = 29, height = 21, units = “cm”, dpi = 300)


The complete script is attached as **Code S3**.

### Conclusion and Outlook

To our knowledge, no single R package specifically targets AM quantification data like Ramf, so it is not possible to compare our package with any other software. We can only compare the package performance to manual operation. Setting up the script and plot customization will take some time at first use, but once the script is ready, it is a matter of seconds to run the script from the beginning to the end for all future data sets (except when customizing the exported plots for each specific data set). A custom script (**Code S4**) has been run 100 times on gridline data and the duration of the execution has been recorded. The average time to run the script top to bottom is 1.047771 s and the standard deviation 0.08366599 s on 100 executions.

As quite some time is needed to master R, Ramf has been designed to facilitate its use for AM quantification data, with few, well documented functions. The user should be able to perform ready-to-publish statistical analyses and graphics with few lines of code at no cost.

We hope the community will drive the future development of Ramf and aim to integrate the Ramf package to develop a graphical user interface (GUI) with Shiny (“InteRamf”) in order to remove the need to learn to use R. With InteRamf, users will be able to produce and quickly save graphical plots and statistical summaries. Before implementation of InteRamf we request the community to try our Ramf package and suggest further improvements on the Ramf github page.

## Data Availability Statement

All datasets generated for this study are included in the manuscript/**Supplementary Files**.

## Author Contributions

MC conceived and developed the Ramf package. DD produced the experimental data set for gridline intersect method, verified the Ramf pipeline with this data set and suggested package function improvements. CG suggested improvements to the data display and output. MC and DD prepared figures; MC, DD, and CG wrote the manuscript.

## Funding

DD was supported by a grant of Valent BioSciences LLC to CG; CG was supported by the Emmy Noether program (GU1423/1-1) of the Deutsche Forschungsgemeinschaft (DFG) during most of the study. The funding agencies had no role or influence on the design or execution of the work.

## Conflict of Interest

The authors declare that the research was conducted in the absence of any commercial or financial relationships that could be construed as a potential conflict of interest.

## Supplementary Material

Figure S1 Substrate selection. Statistical output of **(A)** am_2anova_grid() and **(B)** am_anova_grid() functions to select the optimal substrate for high P mediated AM colonization.

Figure S2 Homoscedasticity plot. Diagnostic plot for checking ANOVA assumption of equal variance.

Figure S3 Q-Q plot. Diagnostic plot for checking ANOVA assumption of normality.

Figure S4 Plant growth in sand at different phosphate regimes. *Lotus japonicus* plants grown in sand at 6 wpi at the indicated phosphate concentrations.

Table S1 Gridline_Sand. Data for total colonization of *Lotus japonicus* roots when grown in sand.

Table S2 Gridline_Sand-Terragreen. Data for total colonization of *Lotus japonicus* roots when grown in a sand terragreen mix (in the ratio 1:1).

Table S3 Gridline_Sand-Calcined clay. Data for total colonization of *Lotus japonicus* roots when grown in a sand:calcined clay mix (in the ratio 3:1).

Table S4 Gridline_substrate. Data for total colonization of *Lotus japonicus* roots when grown in sand, sand-Terragreen and sand-calcined clay. This data set combines Table S1, Table S2 and Table S3.

Table S5 gridData. Root colonization data used for the gridline intersect data set used in the substrate selection experiment (case study 1).

Table S6 trouvelotData. Root colonization data used for the Trouvelot data set used in the strigolactone experiment (case study 2).

Code S1 Substrate selection.R R script used for assessing statistical differences between total root colonization in three growth substrates (case study 1).

Code S2 gridData.R R script used for the implementation of Ramf package for the gridline intersect data set of the substrate selection experiment (case study 1).

Code S3 trouvelotData.R R script used for the implementation of Ramf package for the Trouvelot data set in the strigolactone experiment (case study 2).

Code S4 RscriptForBenchmark.R R script used in benchmarking Ramf use.
