# Supplementary figures and images for: Ramf: An Open-Source R Package for Statistical Analysis and Display of Quantitative Root Colonization by Arbuscular Mycorrhiza Fungi
Source: Front Plant Sci. 2019 Sep 27;10:1184. doi: 10.3389/fpls.2019.01184 (PMC6777641; doi:10.3389/fpls.2019.01184)

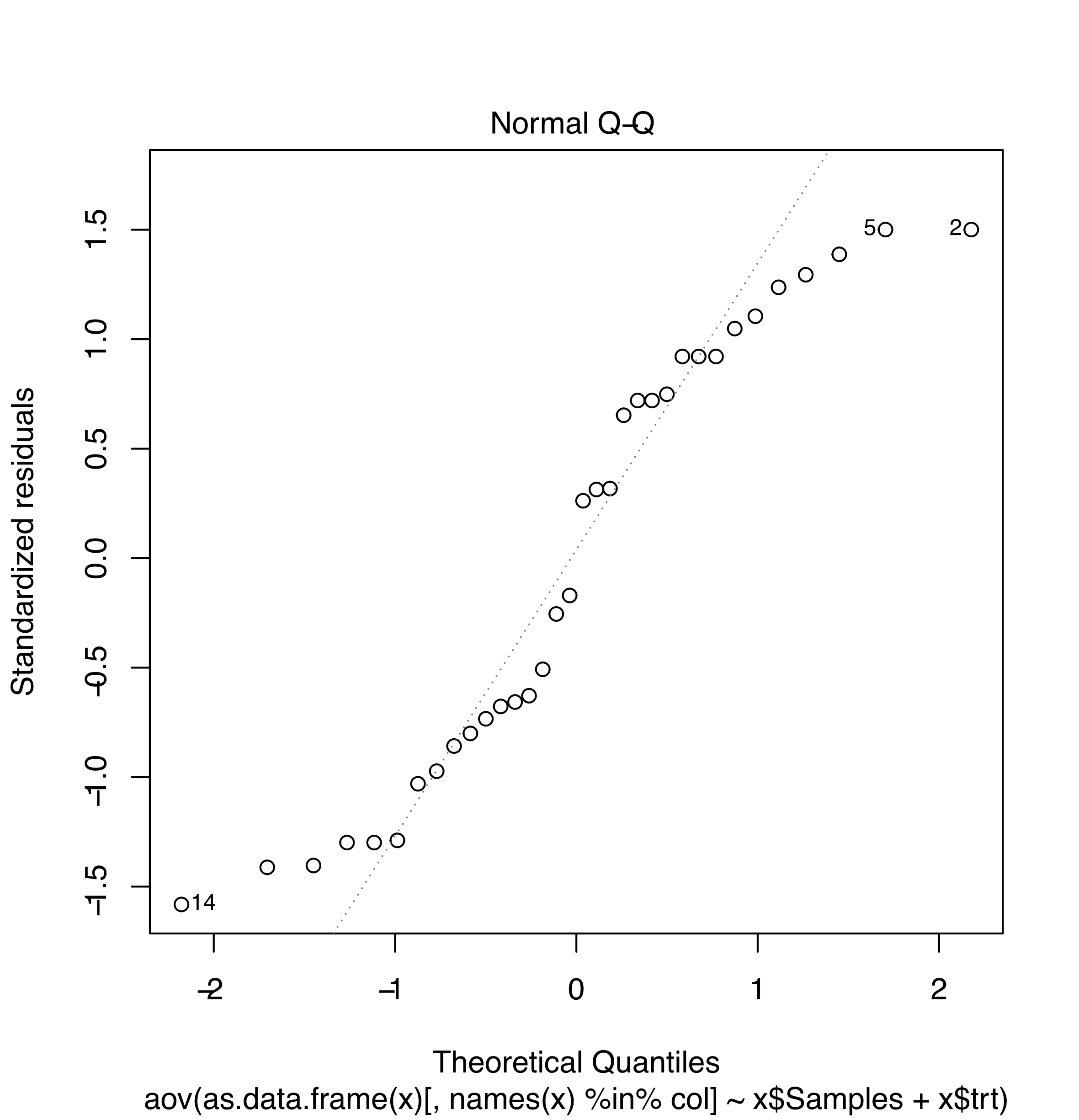

Supplement: Figure S1 — Substrate selection. Statistical output of (A) am_2anova_grid() and (B) am_anova_grid() functions to select the optimal substrate for high P mediated AM colonization. [file DataSheet_1.zip › supplemental figures codes and data/Fig S3.tiff]

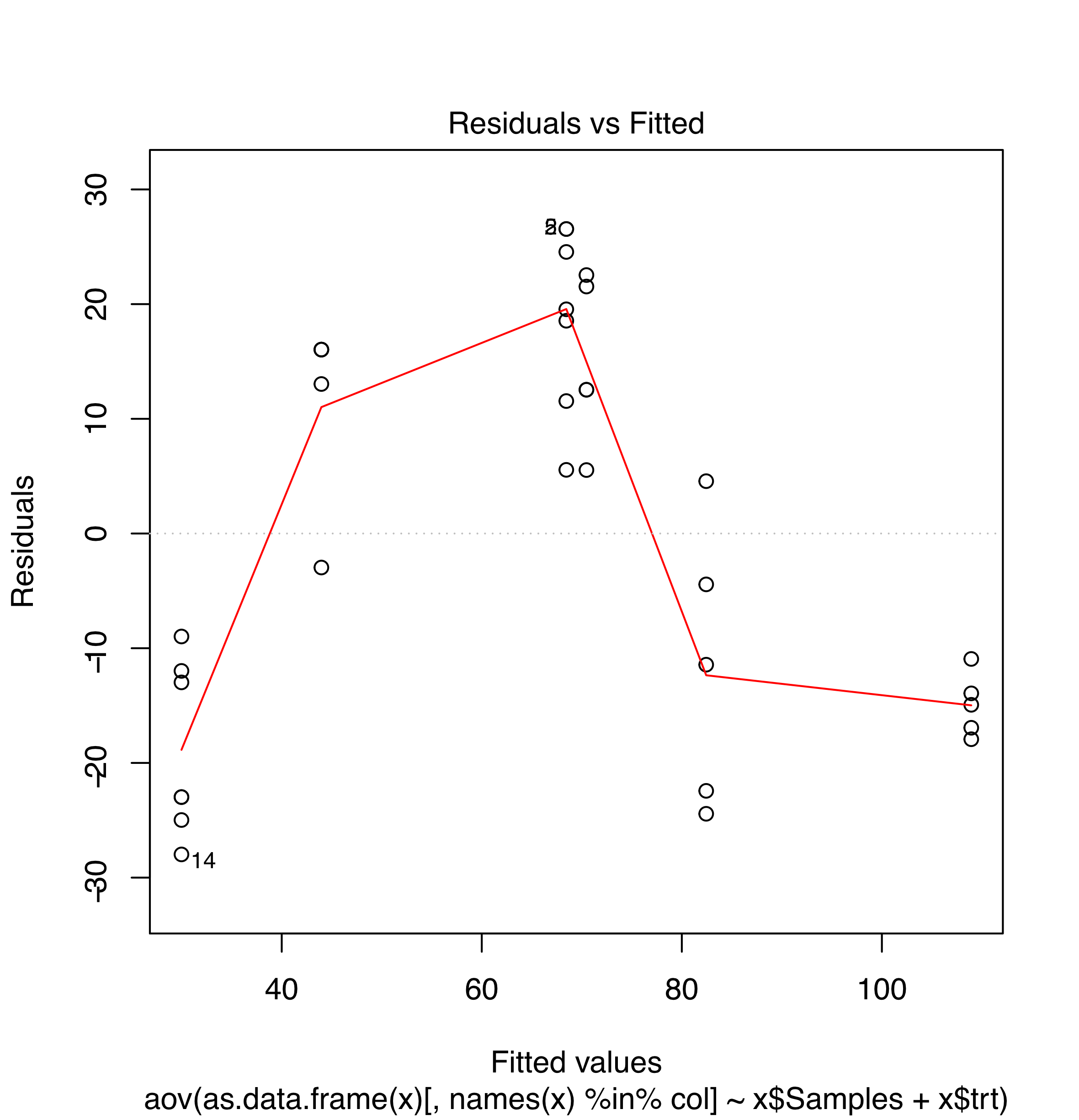

Supplement: Figure S1 — Substrate selection. Statistical output of (A) am_2anova_grid() and (B) am_anova_grid() functions to select the optimal substrate for high P mediated AM colonization. [file DataSheet_1.zip › supplemental figures codes and data/Fig S2.tiff]

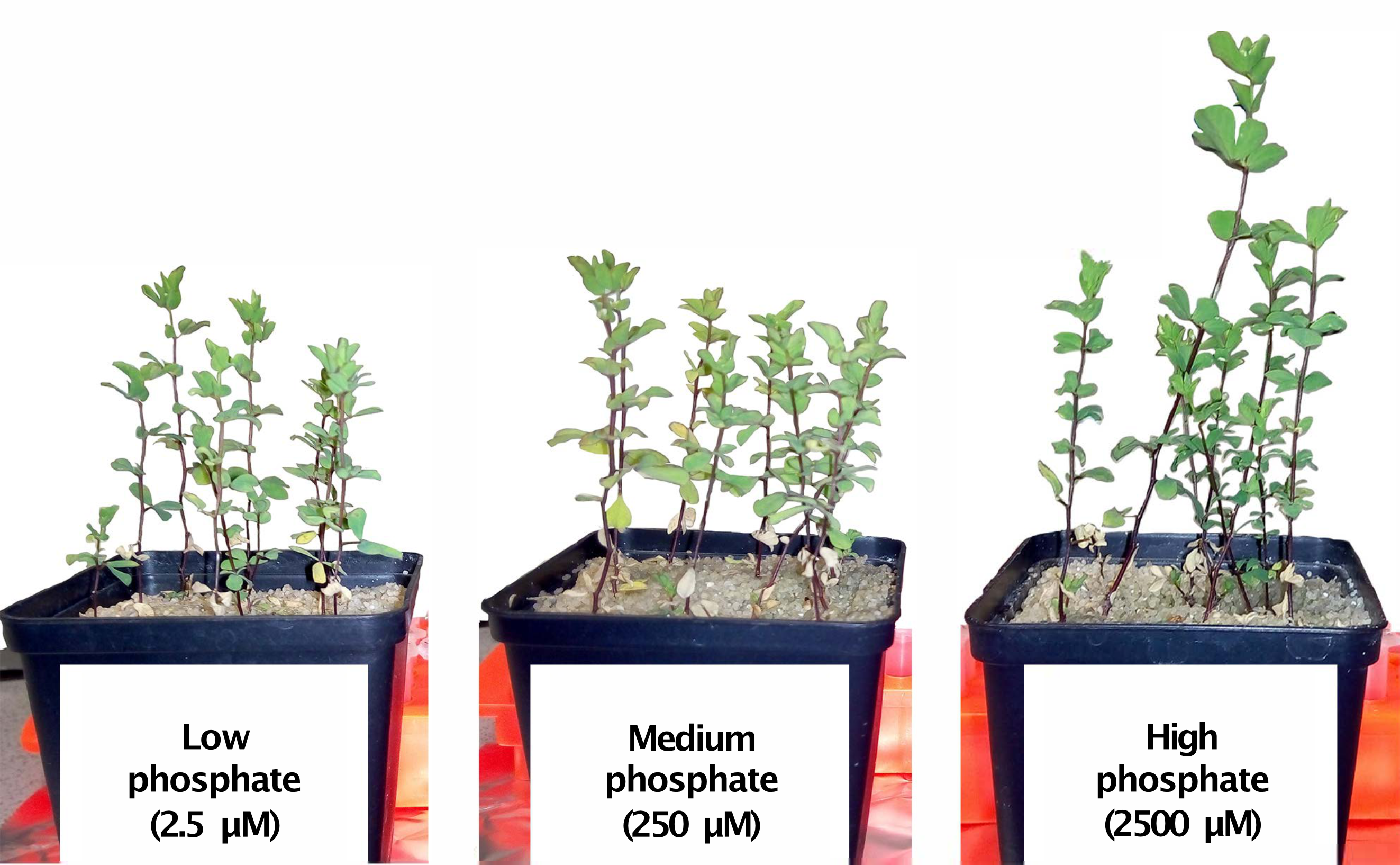

Supplement: Figure S1 — Substrate selection. Statistical output of (A) am_2anova_grid() and (B) am_anova_grid() functions to select the optimal substrate for high P mediated AM colonization. [file DataSheet_1.zip › supplemental figures codes and data/Fig S4.tiff]

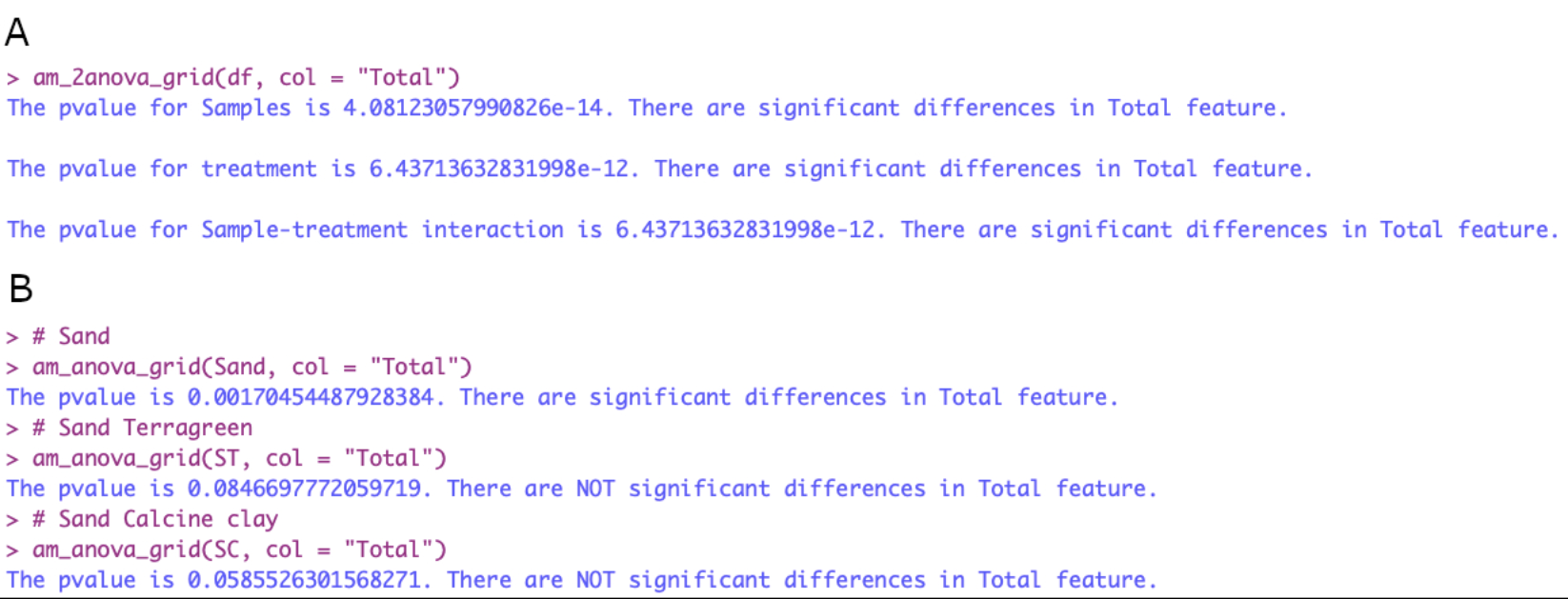

Supplement: Figure S1 — Substrate selection. Statistical output of (A) am_2anova_grid() and (B) am_anova_grid() functions to select the optimal substrate for high P mediated AM colonization. [file DataSheet_1.zip › supplemental figures codes and data/Fig S1.tiff]
